# Observation of correlated electronic decay in expanding clusters triggered by near-infrared fields

**DOI:** 10.1038/ncomms9596

**Published:** 2015-10-15

**Authors:** B. Schütte, M. Arbeiter, T. Fennel, G. Jabbari, A.I. Kuleff, M.J.J. Vrakking, A. Rouzée

**Affiliations:** 1 Max-Born-Institut, Max-Born-Straße. 2A, 12489 Berlin, Germany; 2 Department of Physics, Imperial College London, South Kensington Campus, London SW7 2AZ, UK; 3 Institut für Physik, Universität Rostock, Albert-Einstein-Straße. 23, 18059 Rostock, Germany; 4 Theoretische Chemie, PCI, Universität Heidelberg, Im Neuenheimer Feld 229, 69120 Heidelberg, Germany

## Abstract

When an excited atom is embedded into an environment, novel relaxation pathways can emerge that are absent for isolated atoms. A well-known example is interatomic Coulombic decay, where an excited atom relaxes by transferring its excess energy to another atom in the environment, leading to its ionization. Such processes have been observed in clusters ionized by extreme-ultraviolet and X-ray lasers. Here, we report on a correlated electronic decay process that occurs following nanoplasma formation and Rydberg atom generation in the ionization of clusters by intense, non-resonant infrared laser fields. Relaxation of the Rydberg states and transfer of the available electronic energy to adjacent electrons in Rydberg states or quasifree electrons in the expanding nanoplasma leaves a distinct signature in the electron kinetic energy spectrum. These so far unobserved electron-correlation-driven energy transfer processes may play a significant role in the response of any nano-scale system to intense laser light.

## Abstract (web summary)

In clusters, relaxation of excited atoms can lead to ionization of nearby atoms, a process known as interatomic Coulomb decay. Here, the authors report on a so far unobserved correlated electronic decay process following Rydberg atom generation in clusters ionized by intense near-infrared fields.

## Graphical abstract

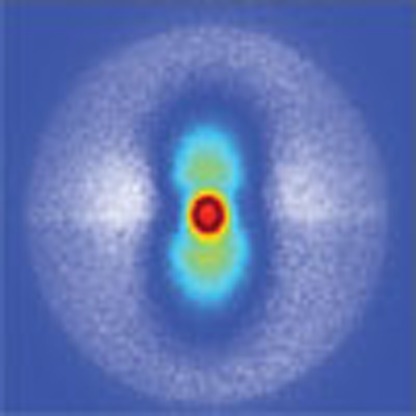

Van-der-Waals bound clusters are tunable in size and composition and often serve as a model for nano-scale systems. When an intense near-infrared (NIR) laser pulse interacts with a cluster, a large number of free charges are generated both by multiphoton ionization and laser-driven electron impact ionization, leading to an efficient heating of the cluster, followed by its expansion and dissociation. Direct and delayed emission of electrons are observed, where the delayed emission encodes the formation and relaxation dynamics of the resulting nanoplasma. Electron confinement in the emerging cluster potential can be overcome by evaporation via electron–electron collisions^1^,^2^,^3^, as revealed by measured thermal electron kinetic energy distributions from clusters ionized by strong light fields. Thus far, other electron-emission processes from nanoplasmas that involve electronic transitions between atomic or ionic bound states and the continuum have been neglected in most experimental and theoretical investigations.

In this paper we report on the discovery of a so far unidentified relaxation mechanism that occurs following the ionization of clusters by an intense (near-infrared) laser. We report the observation of a high-energy peak in the electron kinetic energy spectrum as a signature for the occurrence of a correlated electronic decay (CED) process. The mechanism is interpreted in terms of the formation of low- and high-lying Rydberg states in the expanding nanoplasma^4^,^5^,^6^,^7^,^8^,^9^,^10^. The observed decay channel emerges when these excited atoms relax to their ground state and donate the available energy to a second nearby electron (either quasifree, or itself weakly bound to an atomic core) that can escape the cluster potential. In this respect our mechanism includes the process of interatomic Coulombic decay (ICD)^11^,^12^,^13^ and more specifically one of its variants involving excited states as discussed in refs 14, 15, 16, 17, 18, 19. As the process takes place in a highly dynamic environment, that is during cluster expansion, collisions of excited atoms may play a role. In this case, the mechanism can alternatively be referred to as Penning ionization, which was studied, for example, in He nanodroplets^20^,^21^. We determine a rate for the CED process by monitoring the electron signal depletion induced by a time-delayed probe laser pulse. We observe CED in a number of different clusters and conclude that it is a generic process and a consequence of nanoplasma formation. It is therefore expected to be important in nano-scale systems interacting with laser pulses in different spectral regions.

## Results

### Rydberg atom formation and decay in clusters

**Figure 1:**
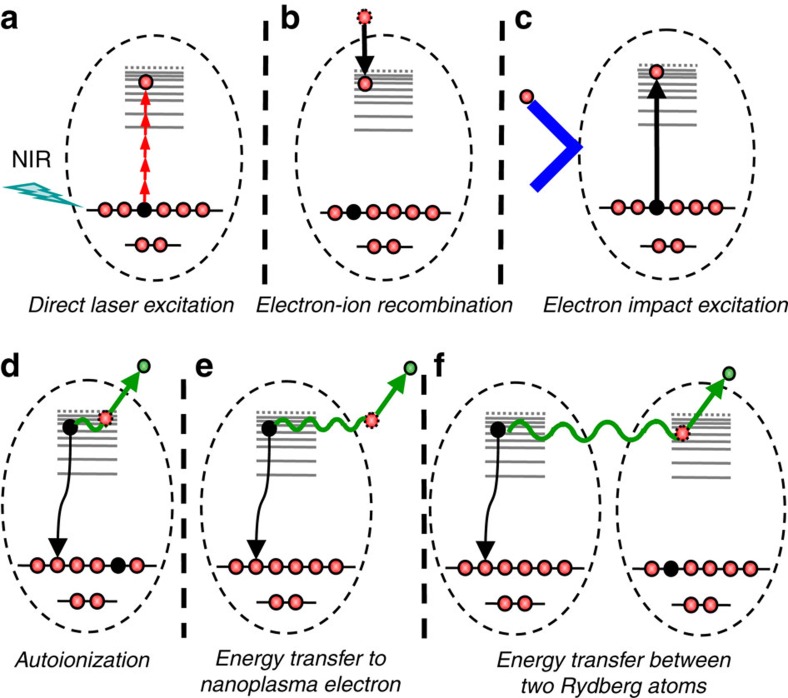
Formation and decay of excited atoms in clusters. Formation of excited atoms can take place (**a**) via direct laser excitation involving a number of NIR photons, (**b**) by electron–ion recombination or (**c**) by electron impact excitation. (**d**–**f**) Mechanisms of CED in clusters, where excited atoms experience a nanoplasma environment and are surrounded by ions, quasifree electrons and excited atoms. Energy exchange can occur (**d**) between two electrons in a doubly excited atom via autoionization, (**e**) from one electron in a Rydberg state to a quasifree electron in the nanoplasma or (**f**) between two electrons in Rydberg states located in different atoms. The energy of the electron emitted via CED can further be changed by interaction with the charged cluster environment.

In clusters exposed to intense laser fields, excited atoms can be formed via direct laser excitation^19^,^22^, electron–ion recombination^4^,^5^,^8^,^9^,^10^ or electron impact excitation^23^,^24^ (Fig. 1a–c). Recent experiments have demonstrated that electron–ion recombination is the dominant mechanism for Rydberg atom formation in clusters exposed to intense laser pulses^8^,^9^,^10^. CED may then take place between two electrons in a doubly excited atom (autoionization)^25^, between a Rydberg electron and a nanoplasma electron or between two Rydberg electrons located in different atoms (Fig. 1d–f). In the latter case, the process represents either Penning ionization (that is involving a collision between the Rydberg atoms) or ICD between Rydberg atoms that form a dimer. When two electrons in Rydberg states are involved in the relaxation process, this is expected to lead to the emission of an electron with an initial kinetic energy *E*_kin_ given by *E*_kin_*=IP−E*_b,1_*−E*_b,2_. In this expression, *IP* is the atomic ground state ionization potential, *E*_b,1_ is the binding energy (positive value) of the electron that donates the energy by relaxing to the ground state and *E*_b,2_ is the binding energy of the electron that gains this energy^14^.

In addition to the processes shown in Fig. 1d–f, energy transfer can also take place via three-body recombination (TBR) or interatomic Coulombic electron capture (ICEC; ref. 26). Therein, an initially quasifree electron recombines with an ion, and the excess energy is transferred in a collision process to a second quasifree electron in the environment (TBR) or to a bound electron, for example in a neighbouring atom (ICEC). These types of electron capture processes may be expected to proceed in early stages of nanoplasma expansion. In fact, previous investigations indicated that electron–ion recombination takes place within the first few ps after cluster ionization^9^, suggesting this as the relevant timescale for TBR and ICEC. As will be discussed below, our new observations of CED are accompanied by significantly slower timescales, and accordingly TBR and ICEC are not included in Fig. 1. We note, however, that TBR and ICEC are examples of electron–ion recombination processes that can contribute to the formation of excited atoms (Fig. 1b), which is a prerequisite for CED. In comparison to TBR and ICEC, which are three-body processes involving one atom and two quasifree electrons or two atoms and one quasifree electron, respectively, energy transfer from an excited atom to a quasifree electron as shown in Fig. 1e may still take place at lower particle densities during an advanced stage of the cluster expansion. Eventually, when the particle density is very low, one can expect that only autoionization of doubly excited atoms (Fig. 1d) or ICD in dimers (or larger fragments; Fig. 1f) contribute to CED.

### Signatures of CED

**Figure 2:**
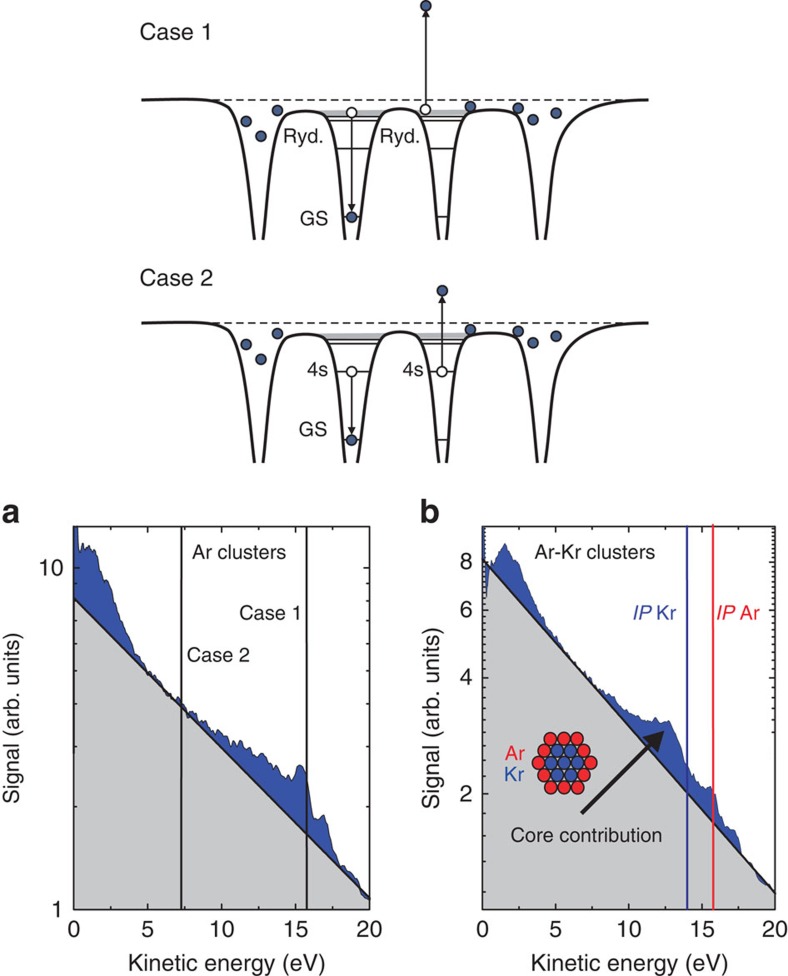
Experimental signatures of CED. (**a**) Electron kinetic energy spectrum after NIR ionization (*I*=1 × 10^14^ W cm^−2^) of Ar clusters with an average size of <*N*>=1,000 atoms, exhibiting a prominent peak structure above a thermal electron kinetic energy distribution characterized by an exponential decay (black line). Case 1: A peak at a kinetic energy close to the ionization potential of atomic Ar (15.76 eV) is attributed to a CED process involving two weakly bound cluster electrons, where one of the electrons relaxes from a Rydberg state to the atomic ground state and transfers its energy to an adjacent Rydberg electron or a quasifree electron. Case 2: marks a decay process involving two Ar*(4s) atoms. Transitions involving other excited states are also possible. (**b**) For mixed clusters with 400 Kr atoms in the cluster core and 950 Ar atoms in the cluster shell (see Methods section), both a peak below the Ar ionization threshold and a peak below the Kr ionization threshold (14.0 eV) are observed in excess of the thermal electron kinetic energy distribution.

Figure 2a displays the electron kinetic energy spectrum resulting from ionization of Ar clusters by intense NIR pulses (see Methods section for details). The spectrum shows a thermal electron kinetic energy distribution characterized by an exponential decay (solid black line in Fig. 2a). In addition, a clear peak emerges at an energy of 15.5 eV, that is slightly below the ionization potential of atomic Ar (*IP*=15.76 eV). To the best of our knowledge, such a peak has never been observed following NIR ionization of either atomic or cluster targets. The peak is only observed in a very narrow parameter range of laser intensities and cluster sizes, and is attributed to CED, where an electron in a highly excited Rydberg state de-excites and transfers its energy to a nearby electron that is in a Rydberg state itself or that is quasifree and bound by the cluster potential.

We note that a tail towards lower kinetic energies (down to ca. 8–9 eV) is present. This tail might indicate the presence of fast decay processes taking place at an early stage of the cluster dissociation that are affected by the presence of the strong cluster potential. Also, CED processes can involve lower excited states. For example, when two Ar*(4 s) atoms (the lowest excited state of Ar with a binding energy of 4.21 eV) are involved, this leads to an initial kinetic energy of the emitted electron of ((15.76 eV–4.21 eV–4.21 eV)=7.34 eV; case 2 in Fig. 2). Transitions may also take place from higher to lower excited states, resulting in electron emission with kinetic energies<4.21 eV. In Fig. 2a, contributions clearly exceeding the exponential distribution, which is assigned to thermal emission, can be observed in this energy range as well. An electron signal above the *IP* including a high-energy peak at 17.2 eV is present that is attributed to CED involving lower excited states (for example, 3d, 4s,...) in the Ar^+^* cation or in the doubly excited Ar** atom. When these states decay to the Ar^+^ ground state, an energy in excess of the first ionization potential is available and can be transferred to an electron in a Rydberg state or a quasifree electron, leading to the creation of an electron with an energy>*IP*.

Non-thermal features close to the *IP* of the respective isolated atoms are observed in all clusters that we have investigated, including Xe_N_ and (O_2_)_N_ clusters (Supplementary Fig. 1), demonstrating the general importance of the investigated decay process. Moreover, in the case of mixed clusters consisting of a Kr core and an Ar shell, two peaks are discernible in the kinetic energy spectrum at energies of about 13 and 15.5 eV (Fig. 2b), reflecting the *IP*s of the two types of atoms. Although the Ar atom fraction is 70% in the mixed cluster, the peak assigned to CED after de-excitation of a Kr atom is dominant, suggesting that the CED process preferentially takes place in atoms originating from the cluster core, where the formation of excited atoms is most efficient^9^,^10^.

### Dynamics of CED

**Figure 3:**
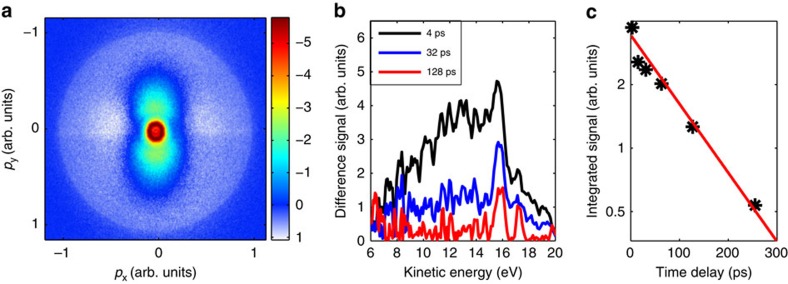
Time-resolved CED data. (**a**) Difference electron momentum map, showing a measurement with a single NIR pump pulse, from which the spectrum obtained in an NIR–NIR pump–probe experiment at a time delay of 8 ps was subtracted. The map illustrates the inhibition of the CED process by the probe. The pump and probe intensities are 1 × 10^14^ and 1 × 10^13^ W cm^−2^, respectively. At low momenta (centre of the momentum map), a negative signal (red and green colour) is observed due to the reionization of excited atoms from recombination by the probe pulse, resulting in an anisotropic distribution. For larger momenta, however, the signal is positive (white colour) due to quenching of the decay process. A ring is visible that resembles the isotropic emission from CED. (**b**) Contributions of electrons from CED at different time delays were obtained by subtracting spectra taken with both pump+probe pulses (with partially suppressed CED contributions) from spectra taken with the pump pulse only (including the observed CED distribution). At an energy of 15.5 eV, and a time delay of 4 ps, 35% of the total electron signal observed in the pump-only experiment is quenched by the probe pulse. (**c**) CED signals integrated between 15 and 16.5 eV, as a function of pump–probe time delay; the red curve is an exponential fit, giving a CED time of 87 ps.

We have studied the rate of the observed decay process by using a moderately strong probe pulse (10^13^ W cm^−2^, that is an intensity where no ionization of neutral clusters is observed) that depopulates the excited state levels and thus quenches the signal from CED. As an example, Fig. 3a shows the difference between two momentum maps measured without and with the NIR probe laser pulse, with an NIR pump–probe delay of 8 ps in the latter case. This difference momentum map is a measure of the electron emission that occurs in an NIR-only experiment for times greater than the delay where the probe pulse inhibits the CED process in the pump–probe experiment. Figs. 3b,c furthermore show the delay dependence of the corresponding difference electron kinetic energy distribution. In Fig. 3c the peak at 15.5 eV decreases exponentially with a time constant of 87 ps when varying the pump–probe time delay from 4 to 256 ps.

**Figure 4:**
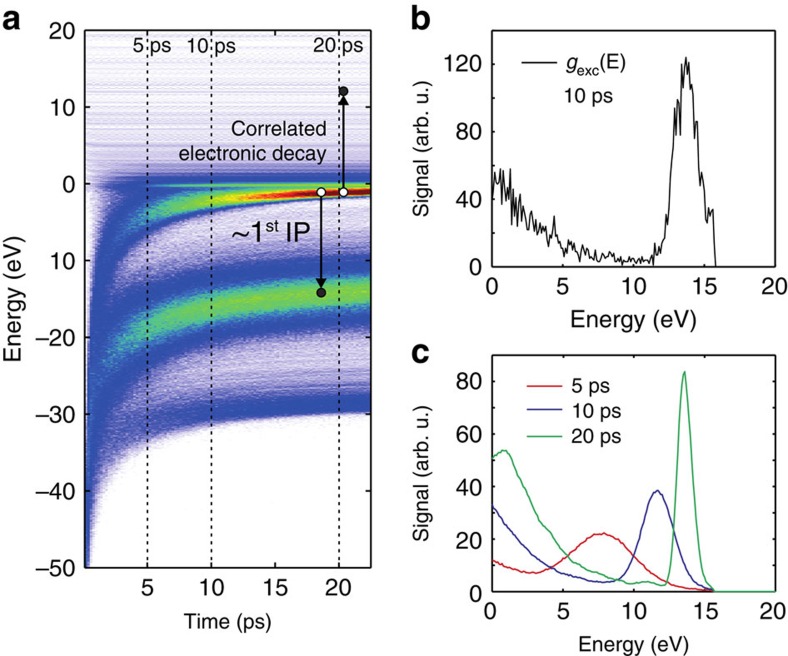
Simulated formation and decay dynamics of weakly bound electrons. (**a**) Evolution of single-particle-energy spectra of activated electrons in Ar_923_ clusters following ionization by 30 fs NIR pulses at an intensity of 1.4 × 10^14^ W cm^−2^. The distribution close to the continuum (*E*=0) represents loosely bound electrons (Rydberg electrons and quasifree electrons bound by the cluster potential), whereas the contributions converging to ∼−15 and −30 eV reflect neutral ground state atoms and singly charged ions resulting from collisional electron–ion recombination reflect electrons being localized to singly or multiply charged ions. Vertical arrows indicate schematically the correlated energy exchange within the CED process. (**b**) Total excess energy distribution *g*_exc_(*E*) of activated electrons, representing the energy that can be released via the decay to the ground state of a nearby singly charged ion at a time of 10 ps. See Methods for a detailed discussion of the treatment. (**c**) Predicted electron contribution from CED at different times after laser excitation. Only for a sufficiently long expansion time of the cluster, the observation of a clear peak close to the IP of atomic Ar can be expected. The contribution at small kinetic energies corresponds to energy transfer processes, where one or both electrons are more deeply bound.

For ICD following inner-shell excitation in dimers using an XUV free-electron laser, a decay time of 150 fs was recently measured^27^. However, in our experiment, CED occurs in an expanding cluster, implying that only decay processes on slower timescales will be visible. The final kinetic energies of electrons that originate from fast decay processes are heavily affected by the cluster potential, see Fig. 4 and the discussion below. Hence these electrons are indistinguishable from the electrons that constitute the thermal background.

For an estimation of CED rates, we will consider the comparably simple case of a dimer that is formed by two excited atoms in the expanding cluster. Note that the binding energies between excited atoms can be in the range of 1 eV^15^, which is much larger than the meV binding energies between neutral ground-state atoms. ICD rates in the case of higher excited atoms formed in the nanoplasma are estimated using the virtual-photon model described in ref. 28. Here, the ICD process is described by the emission of a virtual photon upon the de-excitation of an excited atom. This virtual photon is then absorbed by a neighbouring atom that is ionized. The associated decay width (in atomic units) is given by^14^


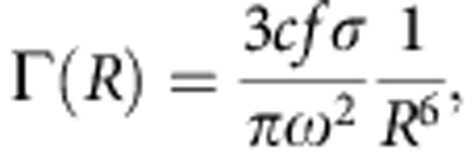


where *c* is the speed of light, *f* is the oscillator strength of the involved transition in the first atom, *σ* is the cross section for ionization of the excited electron in the second atom, *ω* is the energy of the virtual photon, and *R* is the distance between the two excited atoms.

We have performed an *ab initio* calculation^28^ to obtain the lifetimes of the lowest doubly excited states of the argon dimer, Ar*(4s)Ar*(4s) (see Methods section). This gives decay times between 100 and 550 fs for the four lowest states at the equilibrium distance (3.8 Å), or on average about 200 fs. For argon atoms excited to high Rydberg states, the ICD process is expected to take place on much longer timescales. It is known that the cross-sections and the oscillator strengths strongly decrease for higher excited states with respect to the values for *n*=4 (refs 29, 30). For *n*=7, the cross-section is about 10 times lower than for *n*=4 (ref. 29), and the oscillator strength for Ar*(7s) is 10 times smaller than for Ar*(4s) (ref. 30). For even higher excited states, the oscillator strength *f* decreases with *n*^−3^ (ref. 31). In addition, *ω*^2^ is increased by a factor 1.7 when going from Ar*(4s) to Ar*(7s). Based on these changes, the decay time of Ar*(7s)Ar*(7s) is expected to increase by a factor of 170 with respect to the decay time of Ar*(4s)Ar*(4s). This corresponds to decay times of 10s of ps which is consistent with our observations.

Two key requirements must be fulfilled for the observation of a sharp peak close to the first ionization potential. First, a substantial number of neutral Rydberg-like atoms have to be created by the NIR excitation. In our earlier work^9^, we demonstrated that the significant negative signal at low energy in the (pump)−(pump+probe) momentum map in Fig. 3a is due to the reionization of atomic Rydberg states by the probe laser. Second, the energy shift due to the cluster potential must become negligible sufficiently fast to allow the observation of a relatively narrow CED peak.

Support for the fulfillment of these requirements is obtained by quasiclassical molecular dynamics simulations (Fig. 4). Our calculations show that the rapid NIR-induced ionization and cluster potential build-up are followed by cluster expansion. In Fig. 4a, the generation of weakly bound electrons is indicated by the formation of the narrow peak near the continuum threshold (*E*=0) in the electron single-particle-energy spectra. The corresponding timescale of about 10 ps is consistent with the experimental observations in Fig. 3c. The contributions converging to ∼15 and ∼30 eV reflect electrons recombined with multiply charged ions.

Because of the neglect of quantum-mechanical levels, the classical MD propagation cannot capture the CED process directly. Nevertheless, a qualitative prediction of the resulting features in the electron spectra can be achieved under certain additional assumptions. To this end, we consider a strictly bi-electronic energy transfer process involving two activated electrons, where one highly excited electron, either in a Rydberg state or still quasifree, is transferred to the ground state and releases the excess energy to another excited electron. The calculation of the resulting spectra is performed in two steps (see Methods section for details). First, we determined an excess energy distribution, *g*_exc_(*E*), that characterizes the energy release (potential as well as kinetic energy) due to the relaxation of the electron to the ground state. Second, the energy spectrum resulting from the release and transfer of this energy to the other involved electron is calculated from the convolution of the excess energy distribution with the single-particle-energy spectrum of activated electrons. This treatment is based on the assumption that both the single-particle-energy spectrum and the excess energy distribution (see examples in Fig. 4a,b) are representative for the whole cluster.

The spectra from the correlated decay predicted for different times from MD, see Fig. 4c, exhibit a peak-like distribution on the high-energy side that shows the following dynamics. The peak position shifts to higher energies with time and approaches the energy of the first ionization potential. This trend supports the generation of the weakly bound electrons needed for CED and the sufficiently rapid decay of the cluster potential that produces an energy downshift of CED electrons. Further, the decreasing width of the peak with time indicates the formation of a spectrally narrow Rydberg-like population. Though our simplified estimate neither accounts for the rates nor for the detailed state spectrum, the extracted evolution is compatible with the experimentally observed trends, see Fig. 3b. Note that the experimental difference signal would correspond to the temporal integration of the simulated decay spectra starting from the moment of the probe pulse and weighted with a so far unknown time-dependent rate for the recombination efficiency.

### Laser intensity dependence

**Figure 5:**
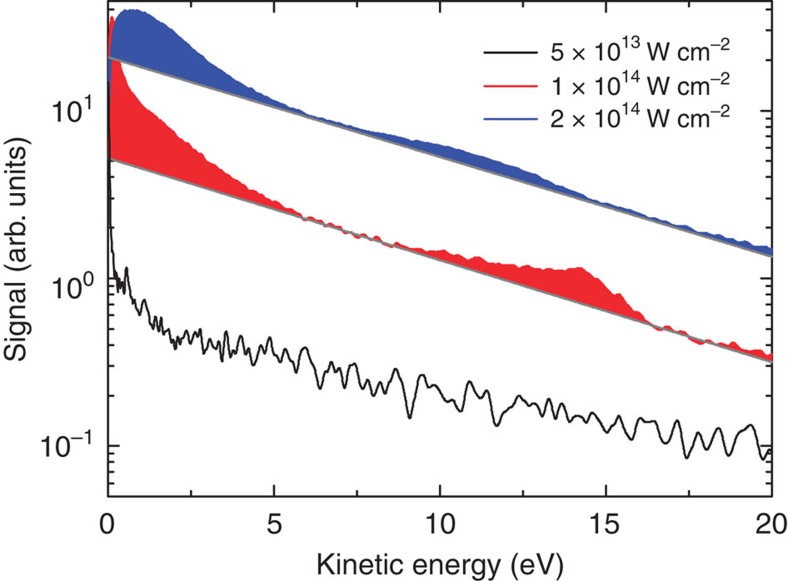
Intensity-dependency of CED in clusters. Angle-integrated kinetic energy spectra (plotted on a logarithmic scale) after ionization of <*N*>=18000 Ar clusters for three different intensities. A peak close to the ionization potential cannot be discerned in the measurement at *I*=5 × 10^13^ W cm^−2^ (black curve), is most prominent in the measurement at *I*=1 × 10^14^ W cm^−2^ (red curve) and shifts to lower kinetic energies in the measurement at *I*=2 × 10^14^ W cm^−2^ (blue curve). Electron contributions attributed to thermal emission are shown by exponential curves for the two highest intensities, highlighting the features attributed to CED.

The CED peak close to the *IP* in the electron kinetic energy spectra is only pronounced in a small range of intensities (Fig. 5). At the lowest NIR intensity tested experimentally (*I*=5 × 10^13^ W cm^−2^), the electron kinetic energy spectrum does not exhibit a peak, since the density of Rydberg atoms is too low. This situation is similar to experiments on clusters with intense extreme-ultraviolet (XUV) pulses using a high-order harmonic generation source^8^, where the density of Rydberg atoms is comparably low and where we have not observed signatures of CED. At an intermediate intensity (*I*=1 × 10^14^ W cm^−2^), a peak from CED appears in the electron spectrum at an energy corresponding to the atomic ionization potential (Fig. 5). When the intensity is further increased (*I*=2 × 10^14^ W cm^−2^), this peak is broadened and shifts to lower kinetic energies, demonstrating that the emitted electrons lose energy that is transferred to other particles in the surroundings by Coulomb interactions (Fig. 4). The observed behaviour at these higher intensities is consistent with investigations on ICD after resonant XUV excitation of He nanodroplets^19^. A similar trend to the one observed in Fig. 5 occurs when the cluster size is varied (Supplementary Fig. 2): In larger clusters the effect of the nanoplasma environment persists for longer time, and the corresponding peaks broaden and shift to lower energy.

## Discussion

CED has escaped observation so far, in spite of a large number of previous studies on intense laser-cluster interactions. One of the reasons is that the CED process only clearly stands out in experimental observables in a very specific parameter range (Fig. 5 and Supplementary Fig. 2). Whereas in the experiment, one can only observe CED processes with decay times in the ps range, the conditions for electron-correlation-driven energy transfer processes are even more favourable on a fs timescale, where the density of charged and excited particles is much higher. Additional processes like TBR and ICEC may play an important role at the early stages of the cluster expansion. CED is of universal nature and, in contrast with previous ICD observations, is not limited to high incident photon energies. Since CED is a consequence of nanoplasma formation, we may also expect it to take place in experiments at free-electron lasers using intense XUV or X-ray laser pulses. We predict the process to be important in a large number of excited states of matter, including biomolecules interacting with intense laser pulses.

## Methods

### Experimental methods

In the experiment, NIR pump and probe pulses were derived from a 50 Hz Ti:sapphire laser system delivering pulses with energy up to 35 mJ and a pulse duration of 32 fs (ref. 32), by making use of a Mach–Zehnder interferometer. The pump and probe pulses were focused into the interaction zone by a spherical mirror with a focal length of 75 mm. Clusters were produced by a piezoelectric valve running at a repetition rate of 10 Hz. A conical nozzle with a 0.5-mm diameter generated the cluster beam, which was then skimmed by a 0.5-mm diameter orifice before intersecting the laser beams at right angles. Control over the cluster size was achieved by changing the backing pressure. The corresponding cluster sizes were estimated via the well-known Hagena scaling law^33^. Mixed ArKr clusters were generated by coexpanding a gas mixture consisting of 95% Ar atoms and 5% Kr atoms. In this case, the cluster size was estimated by evaluating the sizes of pure clusters with the given backing pressures and multiplying the values by 0.95 for Ar and 0.05 for Kr. As a result, the mixed ArKr clusters used in the experiment consisted of 950 Ar atoms and 400 Kr atoms in average. Electron momentum distributions were recorded using a velocity map imaging spectrometer, where electrons ejected from the cluster were accelerated by a static electric field with a field strength of 0.9 kV cm^−1^ towards a multichannel plate/phosphor screen assembly. The two-dimensional projections of the electron momentum distributions were recorded with a charge-coupled device camera, and angle-resolved kinetic energy spectra were obtained by standard Abel inversion algorithms^34^.

### ICD lifetimes

The ICD lifetimes of the spin-singlet two-site doubly excited states of the Ar dimer correlating with the Ar*(3p^−1^4s) excited atoms were calculated using the Fano–ADC–Stieltjes method^35^. The resonance state in this method is represented as a linear combination of bound and continuum parts. These parts together with the coupling between them are computed using an *ab initio* algebraic diagrammatic construction (ADC) scheme for the polarization propagator^36^,^37^. For this purpose we used the ADC(2) extended^38^ method where the electronic Hamiltonian contains the space of 1-hole–1-particle (1h1p) and 2-hole–2-particle (2h2p) configurations and treats the interaction between the 2h2p configurations correct up to first order. The subspace of closed channels is given by all 2h2p configurations, while the subspace of open channels, corresponding to the singly ionized dimer and an outgoing electron, is spanned by the 1h1p configurations. The Stieltjes imaging technique^39^ was employed to ensure the energy normalization of the discretized *ab initio* continuum functions. A correlation consistent cc-pVTZ basis set^40^ augmented by two even-tempered *s*-, *p*-, and *d*-functions, respectively, was used in the calculations. The lifetimes of the states in question at the Ar–Ar interatomic distance of 3.76 Å lie between 100 and 550 fs.

### Cluster simulations

To model the laser-cluster excitation and relaxation, we used quasiclassical molecular dynamics simulations^41^. Starting from closed-shell icosahedral argon clusters, laser-induced ionization was described via Ammosov-Delone-Krainov-tunnelling rates^42^ and Lotz cross-sections for electron impact ionization^43^. Local field-effects on the ionization potentials are then taken into account using the scheme proposed in ref. 5. Both the electrons activated by tunnelling or by impact ionization and the resulting ions are propagated classically under a regularized Coulomb potential whose depth is adjusted to the atomic ionization potential to prevent unphysical recombination. For both, ions and neutral atoms, a binary van-der-Waals interaction is included using a Lennard–Jones potential^44^. Classical collisional electron–ion relocalization is automatically included in the classical propagation^41^. The calculation of CED spectra from a given snapshot of the MD simulations involves two steps. In the first step we determine the excess energy distribution associated with the relaxation of activated electrons to the electronic ground state of the respective nearest singly charged ion. The energy release is calculated from the energy difference of the current state and the state where the respective electron–ion pair has relaxed to the atomic ground state. Note that since both the case of a highly excited Rydberg electron in an atom and the case of a quasifree electron in the vicinity of a singly charged ion are treated in the same way in the classical model, both situations are included in the CED analysis. The predicted CED spectra result from the second step, where the excess energy distribution is convoluted with the single-particle-energy spectrum of activated electrons. We note that the above treatment in principle includes the two decay processes described in Figs. 1e,f and does thus not give preference to either of the decay channels.

## Additional information

**How to cite this article:** Schutte, B. *et al.* Observation of correlated electronic decay in expanding clusters triggered by near-infrared fields. *Nat. Commun.* 6:8596 doi: 10.1038/ncomms9596 (2015).

## Supplementary Material

Supplementary Information Supplementary Figures 1-2 and Supplementary References
