# Supplementary material for: Observation of correlated electronic decay in expanding clusters triggered by near-infrared fields
Source: Nat Commun. 2015 Oct 15;6:8596. doi: 10.1038/ncomms9596 (PMC4634218; doi:10.1038/ncomms9596)
Supplement: Supplementary Information — Supplementary Figures 1-2 and Supplementary References [file ncomms9596-s1.pdf]

Supplementary Figures

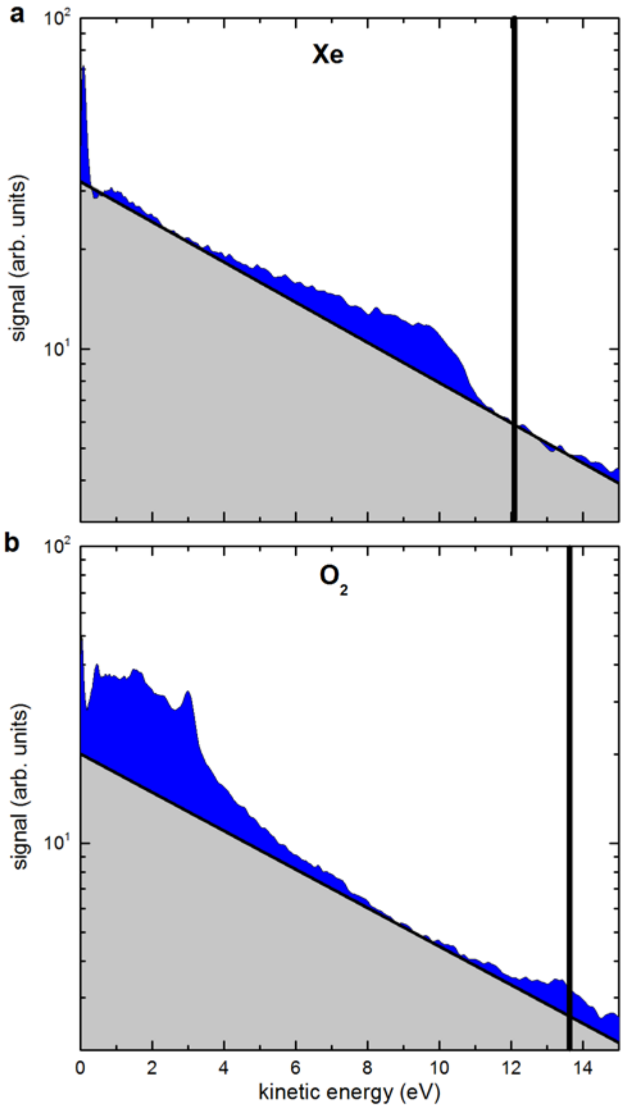

Supplementary Figure 1 | CED for various cluster constituents. (a) Electron kinetic energy spectrum from Xe clusters with <N>=3000 atoms ionized by NIR pulses at an intensity of 5 x 10^{13} W/cm^2. The CED contribution is observed near 10 eV, that is 2 eV below the *IP* of Xe marked by the vertical line. This is explained by the slower expansion of a Xe cluster in comparison to an Ar cluster. Accordingly, the charged environment influences the kinetic energies of emitted CED electrons for longer times in the case of Xe, and thus the CED electrons will experience a larger energy downshift. **(b)** In O_2 clusters with <N>=2400 molecules ionized at an NIR intensity of 9 x 10^{13} W/cm^2, a small peak is visible at the *IP* of atomic O marked by the vertical line. The peaks below 3 eV are attributed to autoionization in isolated atoms [1].

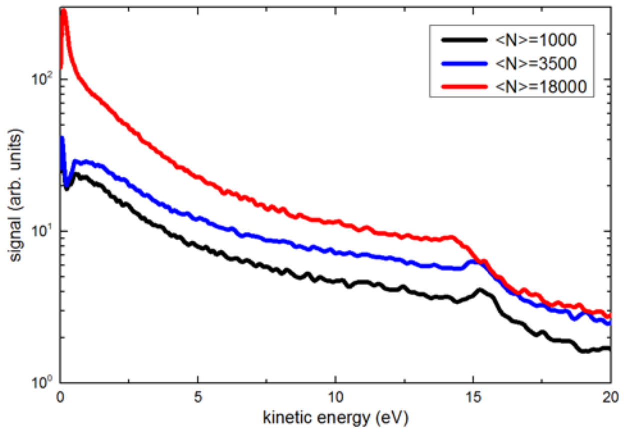

Supplementary Figure 2 | Cluster-size dependent electron kinetic energy spectra. Electron spectra from Ar clusters for different average cluster sizes after irradiation by NIR pulses at an intensity of 1 x 10^{14} W/cm^2. The peak attributed to CED shifts towards lower kinetic energies when the average cluster size is increased from 1000 to 18000 atoms. Similar to the changes of the peak position in the intensity-dependent cluster ionization studies shown in Fig. 5, the shift observed here is associated with the increasing influence of the charged environment, when more ions and electrons are generated in the cluster by the NIR pulse. As energy exchange with the environment becomes increasingly important for larger clusters, the observed peak is broadened as well.

Supplementary References:

[1] Schütte, B., Lahl, J., Oelze, T., Krikunova, M., Vrakking, M. J J. & Rouzée, A. Efficient autoionization following intense laser-cluster interactions. *Phys. Rev. Lett.* **114**, 123002 (2015).
